# The impact of COVID-19 on the practice of Oral and Maxillofacial Pathology in the United States and Canada

**DOI:** 10.4317/medoral.25382

**Published:** 2022-04-14

**Authors:** Vimi Sunil Mutalik, Jasbir Upadhyaya, Mê-Linh Lê, Dieter J Schönwetter

**Affiliations:** 1 BDS MDS MS FRCD(C), University of Manitoba Dr. Gerald Niznick College of Dentistry, Winnipeg, MB, Canada; 2 BDS MSc PhD, Southern Illinois University School of Dental Medicine, Alton, IL, USA; 3 MA MLIS, University of Manitoba, Neil John Maclean Health Sciences Library, University of Manitoba Libraries, Winnipeg, MB, Canada; 4 BTh BA MA PhD, University of Manitoba Dr. Gerald Niznick College of Dentistry, Winnipeg, MB, Canada

## Abstract

**Background:**

The COVID-19 pandemic has significantly disrupted the delivery of healthcare, including oral healthcare services. The restrictions imposed for mitigating spread of the virus forced dental practitioners to adopt significant changes in their workflow pattern. The aim of this study was to investigate the impact of the pandemic on the practice of oral and maxillofacial pathology in two countries in regard to educational activities, and clinical and diagnostic pathology services.

**Material and Methods:**

An online questionnaire was distributed to oral and maxillofacial pathologists in the United States and Canada. The survey was designed by combining dichotomous, multiple choice, and Likert response scale questions. Statistical analysis of the collected data was performed with SPSS software.

**Results:**

Most pathologists, at the time of survey completion, were teaching synchronously, primarily with case-based learning and live lectures. During lockdown, 52.4% and 50.0% of those with trainees expected their residents to show up for clinic- and laboratory-related procedures respectively. The pathologists were most concerned for their residents’ inadequate clinical exposure, future placement, and face-to-face teaching time. About 89.0% pathologists were able to provide emergent care, with 82.4% and 23.5% having performed telehealth consultations and oral biopsy procedures, respectively. During the lockdown, the pathology laboratories for 90.9% of participants received biopsy specimens that predominantly comprised of potentially malignant or malignant lesions. However, a reduction in the number of biopsy submissions was reported.

**Conclusions:**

Given the challenges of the pandemic, oral and maxillofacial pathologists in the United States and Canada successfully continued their pursuits in education, clinical care, and diagnostic pathology services.

** Key words:**COVID-19, digital pathology, oral and maxillofacial pathologists, pandemic, telehealth.

## Introduction

The novel coronavirus disease 2019 (COVID-19) pandemic has resulted in unprecedented challenges for healthcare systems, requiring healthcare practitioners to implement measures to mitigate the spread of virus and to conserve resources. Because of the risk of transmission of the virus through aerosol-generating procedures, dentists were classified as a very high risk category of workers (1). In light of this, the American Dental Association (ADA) and the Canadian Dental Association (CDA), in March 2020, recommended postponement of all elective procedures and restriction of dental care to emergency procedures only. This overnight shift in the workflow pattern had significant implications on patient care, dental education, and the mental health of dental practitioners and students (2,3). The necessary restrictions and sudden closure of academic institutions, instigated the need for adaptation and exploring alternate ways to continue pursuits in educational, clinical, and scholarly activities.

Like other dental specialists, oral and maxillofacial pathologists (OMFPs) introduced dramatic changes in their work pattern. After cancellation of annual conferences due to travel restrictions, the American Academy of Oral and Maxillofacial Pathology (AAOMP), recognizing the importance of continuing education and scientific content, organized virtual meetings in 2020 and 2021. The remote meetings provided continuing education opportunities to an increased number of pathologists, particularly those from outside the United States (4). The American Board of Oral and Maxillofacial Pathology (ABOMP) successfully conducted virtual annual certifying examinations via a secure, live tele-proctored platform both years.

Despite the various challenges posed by COVID-19, the OMFPs continued providing patient care by either switching completely to telehealth or using a hybrid model of a combination of telehealth and in-person consultations (2,5). For effective biosafety practices, work protocols for pathologists and laboratory personnel were modified (6). The need for digital pathology as an alternative diagnostic method was reinforced which allowed remote reporting by pathologists outside their work environment (7). Educational sessions were transitioned to virtual learning in the form of conventional remote classes, discussion of clinical-pathology cases, and other learning activities (8). Research and scholarly activities were greatly impacted during this time of uncertainty. However, the specialists, with their perseverance and dedication, found alternate ways to accomplish their goals (4,9). Despite the rapid evolution of the COVID-19 pandemic and the immense literature published on its impact on dentistry, data on its impact on the practice of oral and maxillofacial pathology remain scarce. Thus, the objective of this cross-sectional study was to capture the experiences of OMFPs in the United States and Canada regarding their education, patient care, and diagnostic pathology services.

## Material and Methods

- Study design

An online survey was shared with OMFPs from March 2021 to May 2021. The questionnaire was developed by two board-certified OMFPs. JU and VM, and psychometrician DS. It was divided into four domains: demographics, didactic training, clinical care, and diagnostic pathology services. The survey comprised of dichotomous, multiple choice, and Likert response scale questions with the option to provide further free text responses for some questions. Furthermore, a few questions were branched into two categories of time, during lockdown and post-lockdown. Lockdown is considered the period when elective care was officially suspended in the United States and Canada (March 2020 to April 2020). The questions were validated by four dental practitioners who provided feedback as to the clarity of each survey item, length of the survey, and any issue in taking the survey. The feedback provided was used to revise and refine the survey. No incentives were offered for participation.

- Survey distribution

The survey was shared with OMFPs through their dental school administrators and professional platforms, Bulletin Board of Oral Pathology (BBOP), AAOMP, and Canadian Academy of Oral and Maxillofacial Pathology and Oral Medicine (CAOMPOM). Only pathologists with an active license participated in the survey.

- Statistical analysis

Responses of participants were evaluated anonymously since no identifiable information was included. Data was collected through the Survey Monkey platform. The demographic characteristics and responses to other questions were recorded in Microsoft Excel, and descriptive analysis was performed using IBM SPSS software. For questions missing responses, only valid responses were included in the final percentage for that question. Thus, the total sample number varied for different questions.

## Results

- Demographic Characteristics

A total of 31 OMFPs completed the online survey. Seven participants [22.6%] were from Canada and 24 [77.4%] from the USA, representing 15.2% and 7.7% respectively of the actively licensed OMFPs. The majority were 55 years or older in age (58.6%; n, 17), with an almost equal gender representation. About 41.4% (n, 12) of the participants had more than 30 years of experience, 24.1% (n, 7) had been in practice for 11-20 years, 17.2% (n, 5) for 21-30 years, and 17.2% (n, 5) for less than 10 years. Twenty participants [74.1%] primarily worked in an academic setting, two [7.4%] in hospitals, and two [7.4%] in private practice. Of the total participants with either part-time or full-time involvement in academia, 20 [74.1%] reported having a residency program at their institute, and were representative of 10 schools. Four [18.2%] were directors of the residency program. Seventeen pathologists provided patient care, either in the school [82.4%] or in private practice [17.6%]. Twenty participants were involved in diagnostic pathology service, 55.0% in school and 45.0% in private practice.

- Impact on clinical activities

During lockdown, workplaces of 88.9% (n, 24) participants were equipped to provide emergent care. About 21.4% (n, 6) were also able to provide elective care. A total of 17 OMFPs [54.8%] were involved in providing patient care. Of these, 82.4% (n, 14) conducted remote- or tele-consultations in this time of uncertainty (Table 1). The most used format was voice calling (over the phone), followed by text format (text messaging or email), and video format (Zoom, FaceTime, Skype, WhatsApp, etc.). A few participants used all three formats (21.4%; n, 3), 42.9% (n, 6) used two formats and 35.7% (n, 5) used one format only (Table 1). In general, 50.0% (n, 15) pathologists performed surgical biopsy procedures. During lockdown, only 23.5% (n, 4) performed oral biopsies (Table 1). When asked about their concern for being unable to consult or follow up patients in the lockdown, 29.6% (n, 8) expressed “a lot” to “a great deal” of concern. Whereas only 11.1% (n, 3) expressed similar concern for being unable to communicate with the patients’ dentist or primary care physician. After re-opening, the number of practice referrals increased for 25.0% (n, 6) participants, decreased for 29.2% (n, 7), had no effect for 25.0% (n, 6), and 20.8% (n, 5) had not determined the effect yet. As part of COVID-19 screening, 47.1% (n, 8) participants asked their patients to use an anti-bacterial mouthwash before examination (Table 1).

- Impact on diagnostic pathology services

Involvement in oral biopsy service was reported by 75.9% pathologists (n, 22; Table 2). The average number of tissue specimens processed annually were: less than 5000, 54.5% labs (n, 12); 5000-10,000, 36.4% (n, 8); and 11,000-15,000, 9.1% (n, 2). During lockdown, 90.9% (n, 20) participants reported receiving biopsy specimens. However, the majority [95.5%] reported a decrease in the number of tissue submissions during this period. The biopsy submissions comprised of either benign lesions only (n, 1), potentially malignant and/or malignant lesions (n, 4), or benign, potentially malignant, and malignant lesions (n, 2). After lockdown, the reported effect on the volume of biopsies was in this order: numbers increased, 45.5% (n, 10); numbers decreased, 40.9% (n, 9); not determined yet, 9.1% (n, 2); and, no effect, 4.5% (n, 1; Table 2).

**Table 1:**
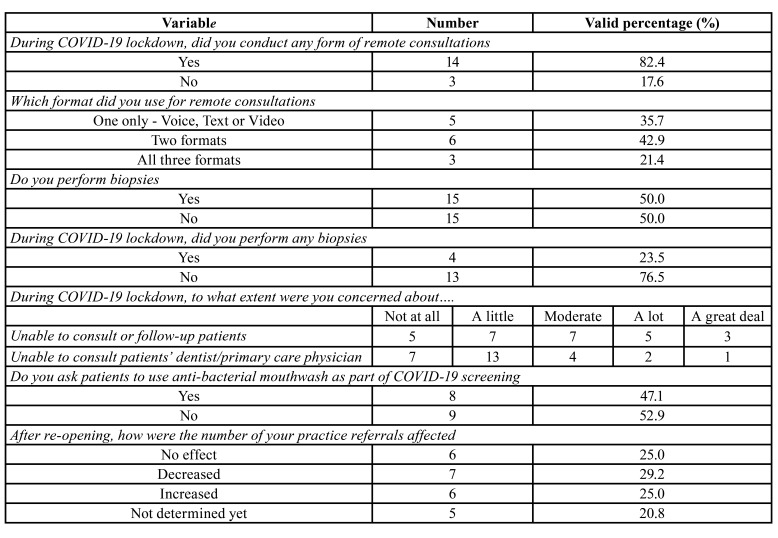
Responses of oral and maxillofacial pathologists for clinical involvement.

**Table 2:**
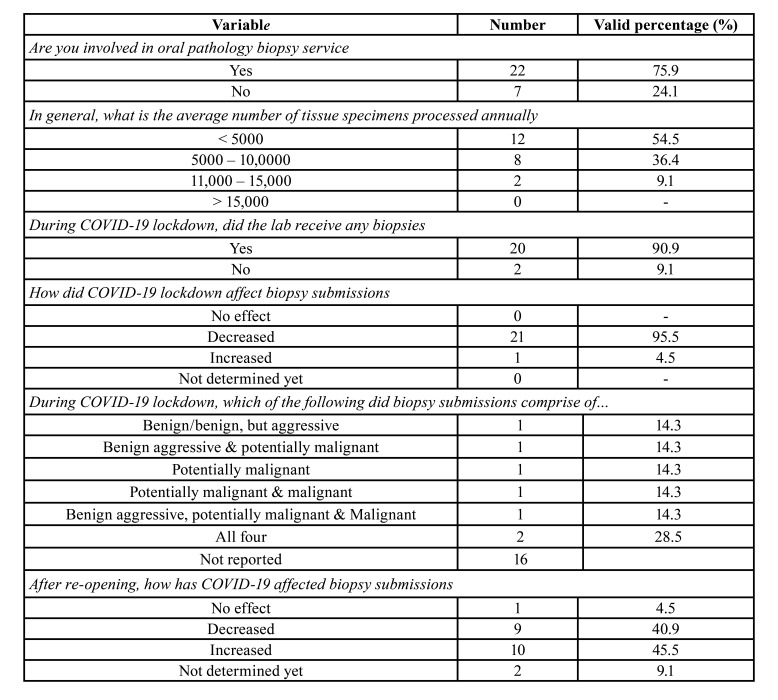
Responses of oral and maxillofacial pathologists for diagnostic services-related questions.

- Impact on educational activities

Of those involved in dental education, the majority (47.4%, n, 11) were using synchronous method of teaching at the time of survey completion. About 25.9% (n, 6) were utilizing asynchronous techniques and only 17.4% (n, 4) were using the hybrid style. Among the various teaching modalities used, case-based learning ranked the highest on a 5-point Likert scale (Mean, 4.18). This was followed by videotaped or recorded lectures (Mean, 3.69), live lectures (Mean, 3.43), problem-based learning (Mean, 3.08), and online articles/journal clubs (Mean, 2.5). In terms of the size of residency program, 33.3% (n, 8) were from programs that comprised of more than 8 residents, 29.2% (n, 7), 5-8 residents, 16.7% (n, 4), 3-4 residents, and 20.8% (n, 5), 1-2 residents (Table 3). During the COVID-19 lockdown, 52.4% (n, 11) and 50.0% (n, 10) of those with trainees expected their residents to show up for clinic-related and laboratory-related procedures respectively. After lockdown, 71.4% (n, 15) of participants had allowed all residents to resume duties with appropriate safety precautions. When asked about their practical engagement with residents for histopathologic slide viewing in general, 63.6% (n, 7) responded to interactive face-to-face engagement, 18.2% (n, 2) interactive online sessions, whereas 18.2% (n, 2) participated equally in both (Table 3). At the time of survey completion, the majority had resumed in-person sessions on multi-headed microscope, with either limited (35.7%; n, 5) or all (35.7%; n, 5) residents present. About 28.6% (n, 4) had not resumed in-person slide review sessions (Table 3). Among the OMFPs concerns for their residents, an inadequate clinical exposure scored the highest on a 5-point Likert scale (Mean, 3.33). The other concerns reported, in decreasing order, were their future placement (Mean, 3.22), inadequate face-to-face teaching time (Mean, 3.11), and timely graduation (Mean, 2.33). A moderate amount of concern was also expressed for receiving new applications for next year (Mean, 2.22; Table 4).

**Table 3:**
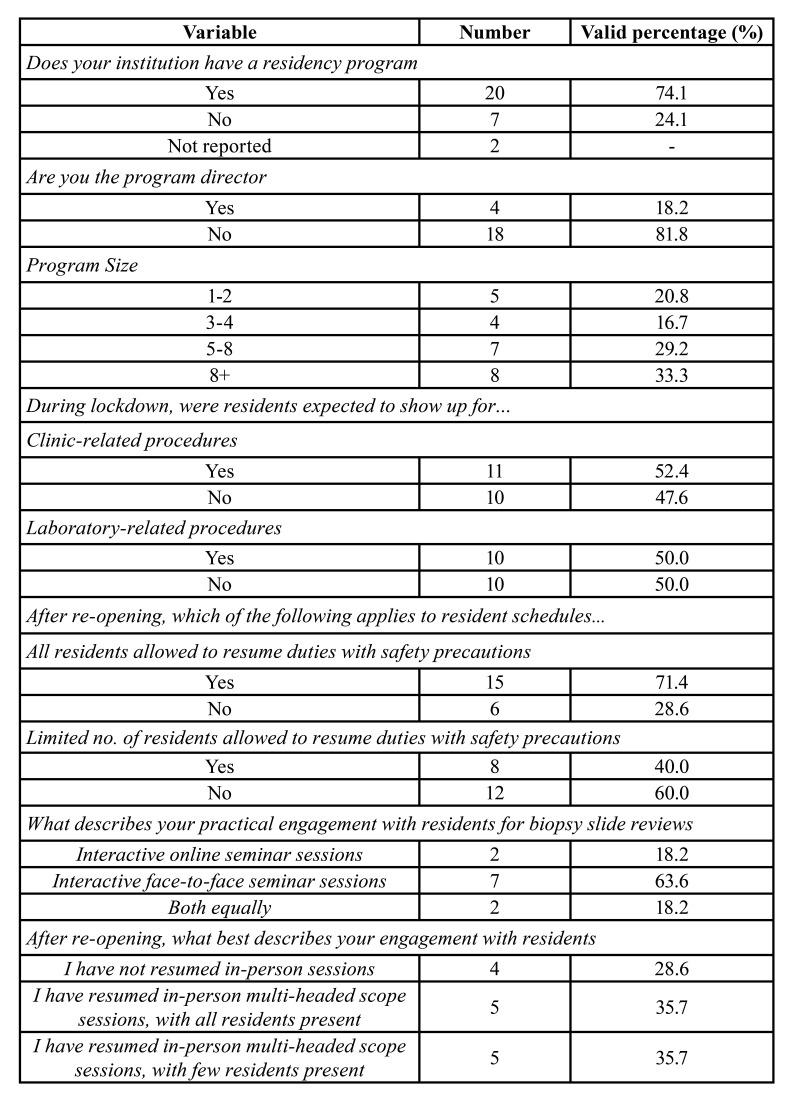
Responses of oral and maxillofacial pathologists for residency-related questions.

**Table 4:**
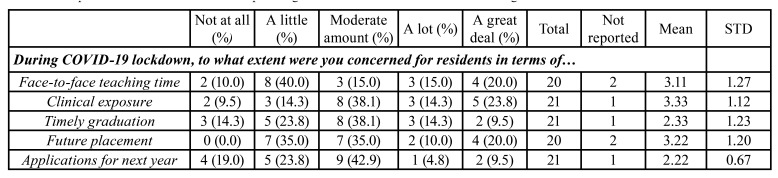
Responses of oral and maxillofacial pathologists for their concern for residents during the lockdown.

## Discussion

This study reports the pandemic-related experiences of 31 OMFPs in the United States and Canada. When universal stay-at-home work orders were established, restricted access to healthcare caused undesirable delays in the diagnosis and management of urgent oral conditions like cancer (10,11). The necessary restrictions imposed resulted in a substantial decrease in outpatient visits in the Oral Medicine and Oncology referral centers, especially during the lockdown period (2,12-14). A large multi-center study from cancer center-based practices documented a 51.5% reduction in patient volume after the stay-at-home orders were established (12). A 60.0% decrease in head and neck cancer referrals in April 2020 was reported by a UK-based report (15). The diagnosis of oral cancer in early stages is one of the most critical factors related to the patients’ survival and severity of the treatment (16). In the pre-COVID-19 era, even with readily available access to healthcare, only less than half of oral cancer patients were diagnosed at an early stage (17), and about one-fourth such patients in the United States experience treatment delays (16). The burden of COVID-19 patients in the hospitals brought cancer care to a halt in many countries. But for diseases that may have a rapid evolution, such as oral cancer, the delay in diagnosis is likely to result in diagnosis of these tumors in more advanced stages. In this regard, it is noteworthy that 21.4% of our participants provided elective care and 88.9% provided emergent care, which we presume may have been for the evaluation of urgent conditions, such as potentially malignant or malignant lesions. And 23.5% performed biopsy procedures during lockdown. Moreover, 52.4% participants expected residents to show up for clinical activities which may very well be for evaluation of urgent conditions. This is in accordance with studies from other institutions where residents, for continuity of clinical education, were allowed to participate in in-person or telehealth consultations (2,18).

The reduction in face-to-face consultations resulted in an increase in the virtual visits (12,14,19). Telehealth provides clinical and supportive care to both established and new patients, and allows effective triage of more urgent cases requiring immediate clinical attention. It has been widely accepted by health professionals (20), effectively incorporated into oncology care (21), and has been well received by both patients and providers (5). In our study, 82.4% oral pathologists provided telehealth services during the lockdown period. The primary format used was voice, over the phone, followed by text format, and video calling with either Zoom, FaceTime, Skype, or WhatsApp applications. Most participants used more than one format for remote consultations. Evaluation of oral conditions such as xerostomia, candidiasis, burning mouth disorder, chronic mucosal conditions, or those requiring pharmacologic intervention would suffice with tele-consultation. However, diagnosis and management, including biopsy, of soft tissue pathologies require face-to-face consultation for a good clinical inspection and tactile assessment. Thus, tele-consultation for such cases may be sub-optimal. A hybrid model of a combination of telehealth and in-person consultation has also been employed (2).

Numerous studies reported a decline in the number of oral biopsies in their laboratories during lockdown (6,22). A study from Brazil reported a reduction of 68.8% in the number of biopsies in 2020 when compared to the same time in 2019 (22). A similar reduction in submission rate of oral malignancies was reported during the lockdown period (23). The same study documented a significant increase in the total number of malignant submissions from January-September 2020, after pandemic restrictions were liberalized. In our study, 95.5% of oral pathologists reported a decrease in biopsy volume during the lockdown period. The specimens received by their respective laboratories primarily consisted of oral potentially malignant lesions, and/or malignant lesions. This alarming decrease in oral biopsies may be attributed to the patients’ fear of being exposed to SARS-CoV-2 infection which led them to avoid seeking health care, even for conditions that should not be ignored.

To facilitate the final reporting of biopsies, pathology laboratories were required to maintain and monitor their workload. The disruptive challenges posed by the pandemic reinforced the need for digital pathology as an alternative reliable diagnostic test. Digital whole slide imaging (WSI) is being increasingly used in many specialties of pathology, including head and neck pathology (24). The memorandum issued by the Centers for Medicare and Medicaid Services (CMS) in March 2020 allowed pathologists to provide remote diagnostic sign-out, even from non-CLIA licensed facilities (7). An increased demand for social distancing in the traditional pathology settings led to an increased use of remote reporting and validation, both for primary diagnosis and second opinion. This change minimized the turnaround time and reduced costs associated with transportation of glass slides. However, its implementation on a large scale is challenging on technical, logistic and financial levels, and may not be a feasible option for small-sized workplaces and those processing a high number of tissue specimens. Most of our participants [90.9%] reported their active participation in biopsy service during the pandemic. However, whether this involved in-office, or remote reporting with use of remote work waiver from CMS was not captured in our study. About 50.0% of participants expected their residents to show up for laboratory-related procedures during the lockdown period. We presume this may have been for grossing of biopsy specimens. Since SARS-CoV-2 strain RNA has been detected in formalin-fixed paraffin-embedded sections of tongue squamous cell carcinoma (25), pathologists and lab technicians adopted safe practice modifications to avoid cross-infection and minimize the risk of spread of COVID-19 in the laboratory while handling specimens.

The COVID-19 pandemic presented significant challenges for didactic training and examination practices globally (2,3,26). Students and educators transitioned to online platforms for didactic learning, continuing education, conferences, and scientific activities. In addition to providing the necessary continued experience and training, the online-learning gave an opportunity for the residents to complete their graduation requirements (2). To enhance learning during the lockdown, many institutions organized clinical and research meetings, and held weekly meetings with students and residents (8). The majority of our participants, at the time of survey completion, were using a synchronous style of teaching, primarily in the form of case-based learning and live lectures, which is in accordance with other studies conducted around the same time (26). For histopathological slide reviews, most pathologists had resumed in-person multi-headed microscope sessions, either with all or a limited number of residents. In contrast, about 28.6% had not resumed in-person sessions. Remote learning may offer a more flexible learning environment, and may be equally or more effective than traditional face-to-face classes (27). However, students in online classes may feel disconnected from their peers and instructors, may need to exercise more self-motivation (28), feel shy participating in discussions, and may have difficulty in accessing or adapting to virtual platform (26). This highlights the increasing need for introducing more interactive ways of learning such as discussion forums, online polls or quizzes, or problem-based learning. During lockdown, OMFPs were most concerned for their residents’ inadequate clinical exposure, followed by their future placement, face to face teaching time and timely graduation.

With COVID-19 shuttering most academic in-person research activities, this has been one of the most affected aspects of graduate education (2). The partial or complete interruptions in scholarly activity negatively affected the morale of oral medicine residents (2). Our study did not capture the pandemics’ impact on research, and it would be difficult to comment on this aspect for the OMFP residents. However, from personal experience we can state that they had parallel experiences to oral medicine residents.

This study has a few limitations. Participation in the survey was voluntary and the sample was based on convenience. The responses of participants were subjective based on their perception of the situation and experiences with the pandemic. The response rate was very low, thus, the demographic data of participants may not be representative of OMFPs across the two countries. This also limited additional data analysis. Since this was a cross-sectional study, a causal relationship could not be evaluated.

In conclusion, we report the COVID-19 pandemic-related experiences of American and Canadian OMFPs in terms of impacting education, clinical care, and diagnostic pathology services. Despite restrictions, and now with surging cases of the Omicron variant, the specialists have been able to continue providing didactic training and clinical care through virtual platforms, while providing diagnostic pathology services by adapting safe laboratory practices.
